# Six-Minute Walking Test Performance Relates to Neurocognitive Abilities in Preschoolers

**DOI:** 10.3390/jcm10040584

**Published:** 2021-02-04

**Authors:** Shelby A. Keye, Anne M. Walk, Corinne N. Cannavale, Samantha Iwinski, Gabriella M. McLoughlin, Linda G. Steinberg, Naiman A. Khan

**Affiliations:** 1 Department of Kinesiology and Community Health, University of Illinois at Urbana-Champaign, Urbana, IL 61801, USA; skeye2@illinois.edu (S.A.K.); gholson@illinois.edu (L.G.S.); 2 Department of Psychology, Eastern Illinois University, Charleston, IL 61920, USA; amwalk@eiu.edu; 3 Neuroscience Program, University of Illinois at Urbana-Champaign, Urbana, IL 61801, USA; cannava2@illinois.edu; 4 Department of Human Development and Family Studies, University of Illinois at Urbana-Champaign, Urbana, IL 61801, USA; iwinski2@illinois.edu; 5 Implementation Science Center for Cancer Control and Prevention Research Center, Brown School, Washington University in St. Louis, St. Louis, MO 63130, USA; gmcloughlin@email.wustl.edu; 6 Department of Surgery (Division of Public Health Sciences), Washington University School of Medicine, Washington University in St. Louis, St. Louis, MO 63130, USA; 7 Division of Nutritional Sciences, University of Illinois at Urbana-Champaign, Urbana, IL 61801, USA

**Keywords:** cardiorespiratory fitness, children, academic achievement, executive function, event-related potentials

## Abstract

This study investigated the relationship between six-minute walking test (6MWT) distance walked and preschool-aged children’s academic abilities, and behavioral and event-related potentials (ERP) indices of cognitive control. There were 59 children (25 females; age: 5.0 ± 0.6 years) who completed a 6MWT (mean distance: 449.6 ± 82.0 m) to estimate cardiorespiratory fitness. The Woodcock Johnson Early Cognitive and Academic Development Test evaluated academic abilities. A modified Eriksen flanker, hearts and flowers task, and auditory oddball task eliciting ERPs (N2, P3) assessed cognitive control. After adjusting for adiposity, diet, and demographics, linear regressions resulted in positive relationships between 6MWT distance and General Intellectual Ability (β = 0.25, Adj R^2^ = 0.04, *p* = 0.04) and Expressive Language (β = 0.30, Adj R^2^ = 0.13, *p* = 0.02). 6MWT distance was positively correlated with congruent accuracy (β = 0.29, Adj R^2^ = 0.18, *p* < 0.01) and negatively with incongruent reaction time (β = −0.26, Adj R^2^ = 0.05, *p* = 0.04) during the flanker task, and positively with homogeneous (β = 0.23, Adj R^2^ = 0.21, *p* = 0.04) and heterogeneous (β = 0.26, Adj R^2^ = 0.40, *p* = 0.02) accuracy on the hearts and flowers task. Higher fit children showed faster N2 latencies and greater P3 amplitudes to target stimuli; however, these were at the trend level following the adjustment of covariates. These findings indicate that the positive influence of cardiorespiratory fitness on cognitive function is evident in 4–6-year-olds.

## 1. Introduction

Fitness is defined as a physiological state of well-being that reduces the risk of hypokinetic disease and enables one to complete the tasks of daily living [1]. One component of fitness, cardiorespiratory fitness, is a vital marker of health during childhood with known benefits for cardiovascular function, metabolic disease risk, and musculoskeletal health [2,3]. A converging body of literature indicates that markers of aerobic exercise capacity, both maximal and sub-maximal, are positively related to children’s academic achievement as well as intellectual and cognitive abilities [1,4]. Mechanisms known to underlie the benefits of cardiorespiratory fitness for cognitive function are thought to involve exercise-induced improvements in brain structure, neurogenesis, synaptogenesis, as well as vasculature [5,6]. Neuroimaging studies using magnetic resonance imaging have revealed that higher cardiorespiratory fitness was related to greater volumes of specific regions of the basal ganglia and hippocampus, and attentional and interference control [7].

Although more research is necessary to comprehensively characterize the influence of cardiorespiratory fitness on the constructs of the brain and cognition, extant evidence suggests a positive relationship among aerobic exercise in children and adults [8,9]. Specifically, greater cardiorespiratory fitness is often associated with positive, albeit small, benefits for cognitive control, an important component of executive function. Cognitive control defines a set of cognitive operations (i.e., working memory, inhibition, and cognitive flexibility) underlying selection, scheduling, coordination, and monitoring of complex, goal-directed processes [1,10]. Working memory is defined as the ability to hold information in mind, manipulate that information mentally, and act based on it. The ability to act based on choice rather than impulse, exercising self-control by resisting inappropriate behaviors, is called inhibition. On the other hand, the ability to quickly and flexibly adapt behavior to changing rules or situations is termed cognitive flexibility [11]. Previous work has demonstrated that cardiorespiratory fitness is associated with greater attentional inhibition [12], working memory [13], and cognitive flexibility [14] among preadolescent children. Brain function has also been directly indexed by examining increased modulation in event-related potentials (ERPs), neuroelectric activations that occur in preparation or response to an event, associated with higher cardiorespiratory fitness [15,16], as well as structural brain differences via MRI [7,17].

Whereas there is an increasing body of literature demonstrating the benefits of cardiorespiratory fitness for cognitive control in childhood, much of this work has focused on children of preadolescent age and older and it is as yet unknown at what point in the developmental trajectory of childhood this relationship emerges. Therefore, comparatively less is known about the influence of cardiorespiratory fitness on academic and cognitive abilities among children of preschool age, i.e., 4 and 5-year-olds. A logical first step toward this end is to examine estimated cardiorespiratory fitness and indices of cognitive function in preschool children, especially as reports suggest that trends toward decreasing physical activity are robust in this population, with nearly half of preschool-aged children not meeting the daily recommended guidelines for physical activity [18]. This presents an important knowledge gap since the development of core cognitive control processes occurs in a protracted manner beginning in early childhood and continues well into early adulthood; thus, clarifying the role of estimated cardiorespiratory fitness on early childhood cognitive control would provide support for earlier interventions to promote optimal cognitive development.

While attempts have been made to further specify the fitness–cognition relationship in preschool-aged children, published studies are scarce with many remaining challenges in methodology and interpretation. Becker, McClelland, Loprinzi, and Trost (2014) investigated a related construct, physical activity, in preschool children, finding that greater levels of active play during recess were associated with higher scores on early academic achievement, specifically in reading and math [19]. Further studies have linked exercise to executive function across childhood (for review see [20]) and non-attentional aspects of cognitive function [21]. While this foundational work is promising, there is a paucity of published work on the relationship between cardiorespiratory fitness and domains of cognitive control, such as cognitive flexibility or inhibitory control, as well as brain-based measures of cognitive function among children aged 5 years and younger.

A promising avenue for such examinations is the use of ERPs. While ERP work is becoming more plentiful in preadolescent samples, it is still rare in preschool-aged children. In samples of children aged 8–11 years old, the N2 and P3b components of the ERP waveform, thought to index response inhibition and the allocation of attention resources, respectively, [22,23] have been studied in inhibitory control paradigms. These studies have shown that higher-fit children exhibit smaller (i.e., less negative) N2 components and larger P3 components compared to their lower-fit counterparts [15,24]. Similar results have been shown in examinations of selective attention, with higher-fit children showing increased P3b amplitudes on a visual oddball task [25].

Furthermore, past research examining cardiorespiratory fitness effects on cognitive control among preschool-aged children has not accounted for children’s degree of excess fat mass or adiposity. Lack of accounting of adiposity is problematic given that excess adiposity negatively impacts cardiorespiratory fitness and has been shown to exert independent and detrimental effects on cognitive control [26,27]. Specifically, children with overweight/obesity completing a six-minute walking test, used to estimate their cardiorespiratory fitness, have exhibited shorter distance, lower heart rate, higher systolic blood pressure, and lower oxygen saturation [28,29]. Pertinent to pediatric populations, excess visceral adipose tissue (VAT), an adipose depot site well recognized for its detrimental metabolic implications, has been shown to predict poorer cognitive function in preadolescent children as well as adults [27,30,31]. Furthermore, dietary intake may also influence cognitive function due to nutritional implications for brain development [32], neuroinflammation [33,34], and the provision of energy [35]. Previous work has shown that school-aged children (7–9-year-olds) with greater adherence to the recommended Dietary Guidelines of Americans, as assessed by the Healthy Eating Index-2005 (HEI-2005), exhibited greater cognitive control, even after adjusting for cardiorespiratory fitness and adiposity [36]. This was consistent with a more extensive study involving over 5000 school-aged children that related dietary quality to greater academic performance [37]. However, to our knowledge, none of the previous studies have accounted for the confounding influence of dietary intake and adiposity when examining the relationship between estimated cardiorespiratory fitness, early academic achievement, and cognitive abilities among school-aged children. Therefore, additional research examining the impact of estimated cardiorespiratory fitness—while accounting for demographic, adiposity, and dietary factors—is necessary to characterize the independent influence of estimated cardiorespiratory fitness on academic skills and cognitive abilities among children of preschool age.

Accordingly, the present work aimed to investigate the relationship between estimated cardiorespiratory fitness, assessed using a six-minute walking test (6MWT) of submaximal exercise capacity, and children’s early academic skills and cognitive abilities, following adjustment of pertinent covariates such as demographic factors, adiposity, and diet quality. Furthermore, using a subsample, we examined the relationship between physical fitness and a brain-based measure of attention in preschool-aged children via an auditory oddball task completed while participants were wearing an electroencephalogram (EEG) cap. We hypothesized that, consistent with previous work in older school-aged children, greater 6MWT distance would be positively and independently associated with children’s academic and cognitive abilities. Furthermore, we anticipated that children with higher fitness would show larger modulations of the P3 and N2 waveforms as indicated by difference waves across task conditions.

## 2. Experimental Section

Participants and Study Design: This was a cross-sectional study whereby males and females between 4 to 6 years old (*n* = 59) were recruited from Eastern Illinois. Flyers using age appropriate language were placed in local schools, community centers, and summer camps, as well as online social media groups, and were used to recruit participants. Children were screened prior to participation and were excluded based on several factors, including the presence of attentional and developmental disorders (Attention-Deficit/Hyperactivity Disorder, Autism Spectrum Disorder, Down’s Syndrome), uncorrected vision, and hearing loss. To determine if these criteria were met, parents reported if a physician had diagnosed their child with any of the aforementioned factors. There were 61 children screened, and all were eligible. All participants provided verbal and/or written assent, and their guardians provided written consent before enrollment in the study. All study procedures were approved by the University of Illinois at Urbana Champaign Institutional Review Board (IRB #16484) and conformed to the guidelines of the Declaration of Helsinki. Study procedures took place over three laboratory visits. During visit 1, 61 participants completed informed assent forms and underwent anthropometric and adiposity assessments. Participants completed the self-paced 6MWT. Additionally, parents/guardians completed informed consent and surveys to report the child’s health history as well as family and child demographic information (i.e., age, sex, level of education, household income). Parents were also given 7-day diet records to complete on behalf of the child completing the study and were asked to return them at the second visit. During visit 2, 60 participants completed the pencil and paper-based neuropsychological battery (Woodcock Johnson Early Cognitive and Academic Development Test (ECAD™)) for assessment of general intellectual abilities. A subsample of participants (*n* = 33) completed a selective attention auditory cognitive task while wearing an electrode cap. This subsample was self-selected in that it consisted of children who tolerated the administration of the EEG cap and completed the task with minimal disruptions to the electrodes. During visit 3, 58 participants completed the modified Eriksen flanker task to assess attentional inhibition. The hearts and flowers task was initially administered during visit 3 (62% of participants); however, due to time constraints on visit 3, the hearts and flowers task was moved to visit 1 (38%) and administered approximately 30 min after the 6MWT.

Although the 6MWT was completed by all 59 children who were enrolled in the study, data may be missing due to participants choosing not to complete some tasks or procedures on the day of testing. Procedures for ECAD™ were completed among 53 participants. Furthermore, 48 and 53 children completed the flanker and the hearts and flowers task, respectively. Diet records were only returned by 55 participants and 53 completed the dual-energy X-ray absorptiometry (DXA) procedure. Lastly, 32 participants completed the auditory oddball task with the electrode cap, whereas usable ERP data was available for 26 participants. A few parents chose not to reveal some demographic information, therefore 51 and 52 participants answered questions regarding race and household income, respectively.

### 2.1. Outcome Measures

Outcome measures considered for potential covariates included demographic information (i.e., age, sex, race), body mass index (BMI), and adiposity, specifically VAT. Sub-maximal exercise capacity was utilized to estimate cardiorespiratory fitness given that this test is well-tolerated by children as young as 3 years old and has high reliability [38,39]. Furthermore, 6MWT has been shown to be predictive of maximal tests of cardiorespiratory fitness (i.e., VO_2max_) among adult samples [40,41] and is shown to have validity across key parameters of maximal workload and oxygen uptake during the VO_2max_ test among children [42,43]. The total distance walked was taken from the 6MWT. For the cognitive tasks, accuracy and reaction time were taken for each trial type. Lastly, for the ERP indices, amplitude and latency were measured.

### 2.2. Procedures

Weight Status and Adiposity Assessment: Participants’ height and weight were measured, without shoes, using a stadiometer (model 240; Seca, Hamburg, Germany) and a Tanita WB-300 Plus digital scale (Tanita, Tokyo, Japan), respectively. Each measurement was taken three times, and the average was used for analyses. BMI-for-age-percentile cut-offs from the Centers for Disease Control (CDC) were used to determine weight status for descriptive purposes [44]. Adiposity was assessed by dual-energy X-ray absorptiometry (DXA) using a Hologic Horizon W bone densitometer (software version 13.4.2, Bedford, MA, USA). VAT was estimated using the standard software measure. This estimated VAT has been shown to correlate (*r* = 0.92; *p* < 0.01) with computed tomography (CT)-determined VAT values [45].

Diet Quality Assessment: Parents completed a 7-day diet record on behalf of their child. Their diet information was analyzed using the Nutrition Data System for Research software (NDSR; Nutrition Coordinating Center, Minneapolis, MN, USA). Healthy Eating Index (Total HEI-2015) scores were derived to determine diet quality. This method has been used in the past for children of the same age group [46].

Six-Minute Walk Test (6MWT): The 6MWT was conducted indoors along a flat and straight walkway using guidelines and procedures previously described [38,47]. Participants were instructed to wear comfortable shoes that allowed them to be physically active. The course length was 20 m, and cones were placed at either end of the walking course to indicate starting and endpoints. The same instructions were given to all children before undertaking the walk test. Participants were tested individually and were provided with standard instructions that the purpose of the test was to find out how far they could walk in 6 min during the allotted time. Instructions for the task included walking back and forth as fast as they could without running from one cone to another and walking around each cone when reaching it. Standard phrases such as “keep going”, “you are doing really well” as well as announcements of time remaining were provided to participants. No comments were made regarding the child’s performance, such as, ‘‘you could go faster’’ or ‘‘slow down’’ although children were reminded of task instructions when needed (e.g., “remember not to run.”). The total distance (m) was recorded and utilized as the variable of interest in the analyses. A portable pulse oximeter (Nonin, Aeromedix, Jackson Hole, WY, USA) was used to assess each child’s heart rate (HR) and oxygen saturation (SpO_2_) at the index finger immediately prior to and following the 6MWT.

Woodcock Johnson Early Cognitive and Academic Development: The ECAD™ is a standardized measure of early academic skills, comprised of 10 tests: seven measuring cognitive abilities and three measuring academic achievements. The ECAD™ produces a comprehensive score, General Intellectual Ability (GIA), as well as two composite scores: Early Academic Skills and Expressive Language. All testing and scoring material was provided by the publisher, including flipbooks, audio recordings, scoring software, and examiner’s manual. This test has been normed and shown to be valid and consistent with other known standardized neuropsychological tests among children [48]. The test was administered according to standardization procedures by a trained member of the research team.

**Figure 1:**
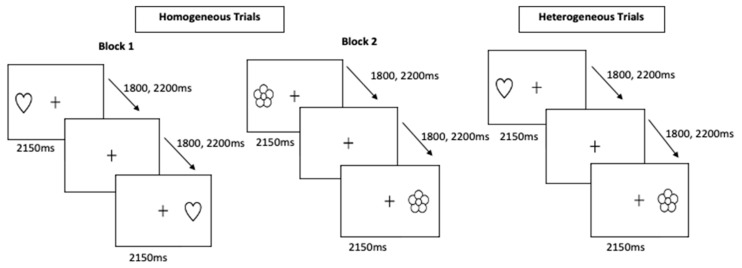
Depiction of the homogeneous and heterogeneous trials of the hearts and flowers task.

Hearts and Flowers Task: A hearts and flowers task, previously shown to be appropriate for children as young as 4 years, was used to assess cognitive flexibility [11]. In all conditions, a white heart or a flower appeared on the right or left of a fixation cross presented on a black computer screen. Participants were asked to indicate the side of the screen either consistent with or in opposition to the stimulus presentation by making a keyboard button press. A different rule set was applied to each stimulus type, such that participants were instructed to indicate the same side of the stimulus appearance for heart stimuli and the opposite side of the stimulus appearance for flower stimuli. The task consisted of three experimental blocks. Participants were given a practice block, where they had to get at least a 70% overall accuracy, to ensure they understand instructions. The first two blocks were considered homogeneous blocks, in which participants were exposed to only one stimulus type (hearts in Block 1 and flowers in Block 2), and thus relied upon only one ruleset. In the third, heterogeneous block, stimuli were mixed, so that participants had to continually switch rule sets eliciting cognitive flexibility, as well as components of working memory and inhibition (Figure 1). Participants completed two homogeneous blocks of 20 trials each (one showing heart stimuli, the other showing flower stimuli) and one heterogeneous block of 40 trials in which the heart and flower trials were mixed. Across blocks, stimuli were presented for 2150 ms with inter-trial intervals (ITIs) of 1800 or 2200 ms. Outcome measurements for this task included accuracy and reaction time for both the homogeneous and heterogeneous trials.

**Figure 2:**
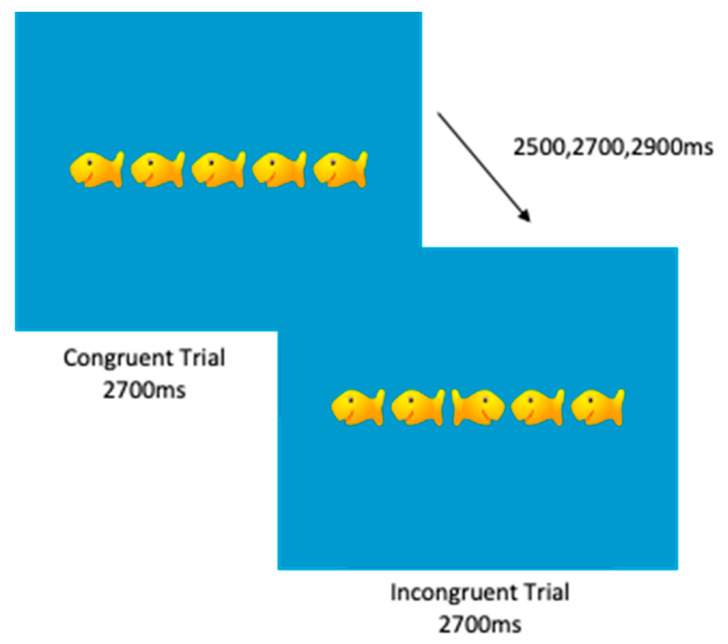
Depiction of a modified Eriksen flanker task for preschool-age children.

Eriksen Flanker Task: A modified version of the Eriksen flanker task was used to assess attentional inhibition [49]. Various versions of the flanker task have proven to be reliable and valid tools to use in preschool-aged children [50], and have been previously employed in children as young as 4 years as well as school-aged children [51]. The flanker task requires children to respond to a centrally presented target stimulus amid an array of 4 flanking stimuli. In the version of the task used in the current study, both the target and flanking stimuli were left- or right-oriented fish, illustrated in Figure 2. The task consisted of congruent trials, in which flanking fish were facing the same direction as the target fish, and incongruent trials, in which flanking fish were facing the opposite direction of the target fish. Participants were given a practice block, where they had to get at least a 70% overall accuracy, to ensure they understood instructions. Successful performance on the incongruent trials, relative to congruent trials, requires cognitive control to selectively attend and respond to the directionality of the target stimulus while suppressing interferences elicited by the flanking stimuli. Congruent and incongruent trials were presented in random order with equal probability based on the congruency and directionality of the target stimulus. Participants completed one block of 50 trials. Stimulus presentation time was 2700 ms with a jittered ITI of 2500, 2700, or 2900 ms. Outcome measures for this task included accuracy and reaction time for the congruent and incongruent trials.

Auditory Oddball Task: A subsample of participants also completed an auditory oddball (AOB) task designed to assess selective attention. The computerized task consisted of 40 high-pitched target tones (1000 Hz) randomly interspersed with 200 low-pitched standard tones (500 Hz). Children were asked to sit as still as possible while wearing an electrode cap and holding a response button box. They were then instructed to hit a button on the box as quickly as possible when they heard the high tone. Tones were played through a speaker system at an approximate decibel level of 60 dB. Participants were given a practice block, where they had to get at least a 70%, to ensure they understood instructions. Outcome measures for this task included accuracy, reaction time, inverse efficiency, and coefficient of variation. Only trials that were correctly responded to were utilized for the behavioral reaction time data. However, ERP data extraction was not behaviorally dependent, and waveforms were extracted for all trials.

ERP Recording: During the AOB task, participants wore a 64 channel Neuro-scan Quikcap to collect encephalographic recording (Compumedics, Charleston, NC, USA). The electrode sites complied with the standard, international 10-10 system. Online, inter-electrode impedances were kept at <10 KΩ; a reference electrode placed between Cz and CPz was used; and the AFz was used as the ground. Continuous data were digitized at a sampling rate of 500 Hz, then amplified 500 times with a direct current to 70-Hz filter, and a 60-Hz notch filter was employed. A Neuroscan Synamps2 amplifier was used. Offline, data were re-referenced to the average of the two mastoid electrodes. A Gratton correction was employed on the midline electrodes to account for noise due to eye blinks. Stimulus-locked epochs were created from −200 to 1500 ms. The −200 to 0 window was used for baseline correction and epochs were filtered using a zero-phase shift low pass filter at 30-Hz. An artifact detection threshold of ±125 μV was employed.

**Figure 3:**
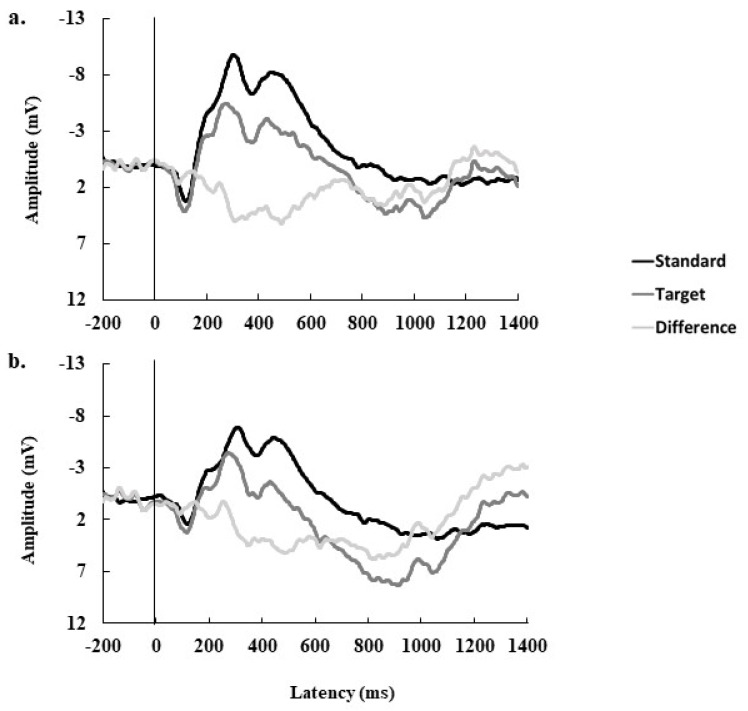
The morphology of the ERP waveforms at electrode at FZ (**a**) and CZ (**b**) are depicted. In each case, the stimulus was presented at 0 ms and is marked with a vertical line. The N2-like component was measured from 150–250 ms post-stimulus (**a**) and the P3-like component was measured from 700–1100 ms post stimulus.

Because ERPs are seldom reported in children within this age range, data were visually inspected to determine time windows and electrodes of interest. To do so, the collapsed localizer method was utilized, in which data from all participants and all conditions were collapsed into a single waveform, which was used for visual inspection [52]. A negativity resembling the prototypical N2 was observed in the Fz electrode within the time frame of 150–350 ms. A later negativity similar to the prototypical P3 was observed in the Cz electrode from 700–1100 ms. The N2 and P3 waveforms are illustrated in Figure 3. Thus, while the terms “N2” and “P3” will be used to reference these components throughout this report, it should be noted that the time windows for these components are later than what is typically seen in older children and adults [53]. The mean amplitude and local peak latency during the time windows specified above were used as metrics of the respective ERP components.

Statistical Analyses: Given that previous research examining the relationship between 6MWT and cognitive function in preschool-aged children is limited [54], a power calculation (G-Power 3.1.9.2) using a small to moderate effect size of *f* = 0.15, α = 0.05, and β = 0.80, determined that a study sample of 55 participants would be sufficient to conduct multiple regressions.

All variables were determined to be normally distributed according to a Shapiro-Wilk test, as well as visual inspection of histograms and Q-Q plots. Initial Pearson’s bivariate correlations were conducted to determine relationships between 6MWT (i.e., distance walked), demographic factors (age, sex, household income), adiposity (i.e., VAT), diet quality (Total HEI-2015), ECAD™, and accuracy and reaction time during the flanker and hearts and flowers tasks. Subsequently, multiple hierarchical linear regression analyses were used to test the variance explained in cognitive outcomes by total distance walked during the 6MWT, following adjustment of the aforementioned demographic and adiposity variables. The significance of the change in the *R*^2^ value between the 2 steps was used to judge the independent contribution of distance walked during the 6MWT to variance in cognitive task performance, beyond that of step 1 or covariates. Given previous work among older children indicating that acute exercise has immediate post-exercise benefits for cognitive control, the task performance was compared based on the day of administration, i.e., visit 1 (after 6MWT) or visit 3 to account for any influence of the 6MWT on the hearts and flowers task performance. Statistical significance was set at *p* *=* 0.05. Statistical analyses were conducted in SPSS version 24 (IBM, Chicago, IL, USA). Given the abundance of literature among older children indicating a positive relationship between cardiorespiratory fitness and cognitive control (for review see [1]), we employed one-tailed statistical tests, consistent with the directional hypothesis.

ERP subsample analysis: Within the subsample of children who completed the auditory oddball task, the relationship between physical fitness and the N2 and P3 components of the ERP waveform was assessed. The relationship with fat (VAT) was not assessed, as the inclusion of the VAT variable would further decrease the size of the subsample. Rather, the relationships between the neuro-cognitive indices, 6MWT performance, and demographic variables were explored via Pearson’s bivariate correlations, with subsequent partial correlations controlling for only age and sex, the two most salient demographic characteristics. Due to the directional nature of the ERP hypotheses, a *p*-value of 0.10 was used as a cutoff to determine moderate statistical relationships.

## 3. Results

### 3.1. Main Sample

Participant Characteristics and 6MWT Performance: Participant characteristics of the main sample are summarized in Table 1. Fifty-seven percent of the study sample was comprised of males. A majority of participants (70%) were of healthy weight status based on BMI-for-age %ile. All children completed the 6MWT. Distance walked during the 6MWT ranged from 240 to 600 m. There were no significant differences between males and females (*p* = 0.68) in the distance walked. Based on previous normative values, the average distance walked among 4-year-olds (431.9 m ± 75.4) fell between the 75th and 90th %ile for age for the 6MWT [39] while the mean value for the 5-year-olds (466.3 m ± 87.5) fell above the 90th %ile for age. There was a significant difference between pre-test (96.2 bpm ± 21.7) and post-test (132.3 bpm ± 24.2) heart rate (*p* < 0.01). However, there was no significant difference between pre-test (96.8% ± 4.7) and post-test (96.6% ± 3.9) oxygen saturation (*p* = 0.84).

Bivariate correlations: Bivariate correlations are provided in Table S1 in Supplementary Materials. Age was associated with distance walked (*r* = 0.29, *p* = 0.01), homogeneous accuracy (*r* = 0.45, *p* < 0.01) and reaction time (*r* = −0.39, *p* < 0.01), and heterogeneous accuracy (r = 0.50, *p* < 0.01) during the hearts and flowers task. Additionally, age was correlated with congruent reaction time (*r* = −0.24, *p* = 0.05) and incongruent accuracy (*r* = 0.26, *p* = 0.04) during the flanker task. Sex (females coded as 0 and males coded as 1) was correlated with congruent accuracy (*r* = 0.31, *p* = 0.02), congruent reaction time (*r* = −0.25, *p* = 0.05), and incongruent accuracy (*r* = 0.37, *p* = 0.01). Diet quality, i.e., Total HEI-2015 was correlated with expressive language (*r* = 0.29, *p* = 0.02). BMI-for-age %ile was inversely related to expressive language (*r* = −0.27, *p* = 0.03). VAT was inversely correlated with estimated early academic skills (*r* = −0.28, *p* = 0.03), and expressive language (*r* = −0.26, *p* = 0.04). There was no significant correlation between household income and any other demographic or cognitive task variables (*r* = −0.18 to 0.14, *p* = 0.11 to 0.41). Additionally, there was no significant difference between participants based on hearts and flowers task administration, i.e., visit 1 vs. visit 3 across all task outcomes (*p* = 0.06 to 0.24). Therefore, the hearts and flowers task order was not adjusted for in subsequent analyses.

Distance walked was positively associated with all three clusters of the ECAD™: GIA (*r* = 0.25, *p* = 0.04), Early Academic Skills (*r* = 0.30, *p* = 0.02), and Expressive Language (*r* = 0.25, *p* = 0.04). Distance walked was inversely correlated with incongruent reaction time (*r* = −0.26, *p* = 0.04) during the flanker task. Homogeneous hearts and flowers task outcomes that correlated with distance walked included accuracy (*r* = 0.37, *p* = 0.01) and reaction time (*r* = −0.28, *p* = 0.02). Distance walked was also correlated with accuracy (*r* = 0.40, *p* < 0.01) but not reaction time (*r* = −0.04, *p* = 0.39) during the heterogeneous or mixed hearts and flowers trials.

Regression Analyses: Linear regression analyses are described in Table 2. The positive relationship between distance walked during the 6MWT and the ECAD™ remained significant for GIA and Expressive Language after adjustment of VAT and HEI-2015. For the Flanker task, there was a positive relationship between distance walked and congruent accuracy following adjustment of sex and GIA, although, distance walked was not related to congruent reaction time. There was also a negative relationship between distance walked and incongruent reaction time on the flanker task. Lastly, distance walked was positively associated with homogeneous and heterogeneous trial accuracy on the hearts and flowers task following the adjustment of age and GIA. Depictions of bivariate correlations of the significant outcomes from the regression analysis are in Figure S1 of the Supplementary Materials.

### 3.2. ERP Subsample

For the subsample with ERP data, participant characteristics are depicted in Table 3. Whereas 32 participants completed the auditory oddball task while wearing the electrode cap, only 26 participants yielded useable data. Participants were excluded if they had fewer than five useable ERP trials (*n* = 4) or if their ERP data were considered an outlier based on the ±3 standard deviation rule (*n* = 2). The children who were not included in the subsample were children who refused the cap, who continually touched and manipulated sensors on the cap, or who were unable to remain sufficiently stationery for recording once the cap was placed. Participants’ behavioral performance indicated that children generally understood the task (M target accuracy = 75%). Bivariate correlations between demographic characteristics, fitness measures, and neurocognitive measures were examined. Age and sex were inversely correlated (although sex only moderately so) to the reaction time for target stimuli, with older children and boys responding more quickly to targets. Age was also inversely related to mean amplitude difference for both N2 and P3, suggesting that older children showed less differentiation in their neural responses to standards compared to targets. There were no significant relationships between performance on the 6MWT and the behavioral metrics of the cognitive task.

Subsequent partial correlations were run to investigate the relationships between sub-cardiorespiratory fitness and neuro-cognitive measures while controlling the variance due to age and sex. The partial correlation table is depicted in Table 4. The analysis indicated that when co-varying age and sex, 6MWT distance was moderately related to target reaction time (*r* = 0.388, *p* = 0.061) and P3 mean amplitude difference (*r* = 0.361, *p* = 0.083). No significant relationships were found between 6MWT performance metrics for the N2 waveform.

As an exploratory secondary analysis, the values for N2 and P3 amplitude and latency for target and standard trials were submitted separately to partial correlation analyses, accounting for variance due to age and sex. This was done to aid in the interpretation of the relationship between fitness and modulation. The results of this analysis showed a moderate negative relationship between 6MWT distance and N2 latency for target trials (*r* = −0.392, *p* = 0.058). For P3, there was a moderate positive relationship between distance and P3 amplitude for target trials (*r* = 0.370, *p* = 0.075). No other relationships with fitness and ERP indices attained marginal significance (all *p*’s ≥ 0.10).

## 4. Discussion

The positive relationship between cardiorespiratory fitness and cognitive function, especially cognitive control and academic achievement, has been well studied in preadolescent children [1]. However, similar studies in younger children remain limited. This study aimed to explore the potential relationship between 6MWT distance walked and early academic skills and neuro-cognitive function in preschool-aged children (4–6 years old). The results showed significant associations between 6MWT distance walked and GIA and Expressive Language, congruent accuracy and incongruent reaction time during the flanker task, and homogeneous accuracy and reaction time and heterogeneous accuracy during the hearts and flowers task, even after adjusting for diet, adiposity, GIA, and demographic factors. Furthermore, data from a subsample of children who completed a selective attention task suggested a relationship between 6MWT distance walked at the modulation of neuro-cognitive metrics. These results are consistent with previous work conducted in both preschool-aged and preadolescent children indicating that the potential for cardiorespiratory fitness to influence cognition is evident in children as early as 4 years old [15,54].

Our results from the ECAD^TM^ revealed significant positive relationships between 6MWT distance walked and GIA and Expressive Language, independent of VAT and HEI-2015. These relationships indicate that preschool children exhibiting greater estimated cardiorespiratory fitness, based on their 6MWT distance walked, may display better performance in skills related to academic achievement. The association between cardiorespiratory fitness and academic achievement has been studied extensively in school-aged children; however, knowledge in preschool-aged children is limited. In a systematic review examining physical activity, fitness, cognition, and academic achievement, the authors found 20 studies examining students from 2nd to 9th grade that reported positive associations between the children’s fitness and their academic achievement [1]. These findings, along with ours, suggest that the relationship between academic achievement and estimated cardiorespiratory fitness can be observed before children enter school. Potential mechanisms contributing to these relationships have been demonstrated in studies on preadolescent children investigating changes in brain structure [55]. Chaddock-Heyman et al. (2015) showed that children with higher cardiorespiratory fitness had decreased gray matter thickness in various portions of the cortex and had superior arithmetic performance. These changes in brain structure and function have been associated with cognitive control and academic achievement in preadolescent children [56]. Considering the results of the present study and these underlying mechanisms demonstrated in preadolescent children, studies investigating brain structure and function in preschoolers are necessary to determine if these same mechanisms are occurring in preschool-aged children.

The results of the flanker task showed a significantly positive relationship between 6MWT distance walked and accuracy on the congruent trials independent of sex, age, and GIA. Interestingly, a significantly negative relationship was found between 6MWT distance walked and incongruent reaction time, but not congruent. Therefore, the influence of estimated cardiorespiratory fitness appears to be evident, particularly when attentional inhibition demands are increased. Similar relationships have been found previously in children of the same age group. For example, Niederer et al. (2011) examined the cross-sectional and longitudinal relationship of cardiorespiratory fitness with spatial working memory and attention in preschool children. Estimated cardiorespiratory fitness, assessed using a 20 m shuttle run test, was positively associated with attention at 5 years of age and was predictive of attentional abilities 9 months later [54]. These relationships are also consistent with studies done in preadolescent children. A study comparing high vs. low fit children ages 8–11 years old found that higher fit children had increased response accuracy in comparison to lower fit children [24].

Cognitive flexibility is another aspect of cognitive control that is influenced by cardiorespiratory fitness. Our results from the hearts and flowers task revealed positive associations between 6MWT distance walked and accuracy in the homogeneous and heterogeneous trials. Thus, preschool age children with higher estimated cardiorespiratory fitness may exhibit a greater capacity to adapt to changing rules or situations in comparison to preschool age children with lower estimated cardiorespiratory fitness. Interestingly, unlike the flanker task performance, the influence of greater 6MWT distance was evident for both the homogeneous as well as the heterogeneous task conditions, suggesting the findings were independent of increasing task demands. Nevertheless, similar to the flanker task, this positive relationship between fitness and cognitive flexibility is also observed in preadolescent children. Pontifex et al. (2014) reported higher cardiorespiratory fitness relating to better response accuracy for the heterogeneous trials of the switch task in preadolescent children ages 7–10 years old [14]. Given our findings and previous research, it appears that the relationship between estimated cardiorespiratory fitness and cognitive control, specifically attentional inhibition and cognitive flexibility, can be observed in early childhood and continue to be observed throughout the preadolescent stage of childhood. Another study on preadolescent children conducted magnetic resonance imaging and observed that children who had higher cardiorespiratory fitness exhibited better performance on the flanker task coupled with a larger dorsal striatum, a region in the basal ganglia involved in cognitive control [7]. Studies involving imaging would provide additional information on whether preschool children also display structural and functional differences in brain regions that support cognitive control.

The results of the subsample analysis indicated that there was no relationship between 6MWT performance and N2 modulation, although it did suggest that children with greater 6MWT distance elicited earlier N2s for target stimuli. This finding is not surprising, given that the N2 has been implicated as a fitness-related component in older, preadolescent children. Pontifex et al. (2011) examined the N2 in relation to high and lower cardiorespiratory fitness in preadolescent children (mean age = 10) and, likewise, found that higher-fit children showed smaller and earlier N2s compared to lower fit children on task-relevant stimuli [15]. However, because of our small sample size, this finding should be interpreted with caution, our data suggest that higher fitness could co-occur with faster neural processing speed in preschool children.

Our data also showed that children with greater 6MWT distance had greater modulation of the P3 component. The follow-up analyses suggested that this effect was driven primarily by larger P3s elicited for target trials amongst those who performed better on the 6MWT. This result is also consistent with prior findings in preadolescent children, which have consistently shown the same pattern of results in both tasks of attentional inhibition [15,24,57], as well as selective attention [25,58]. Chang, Tsai, Chen, and Hung (2013) showed a facilitating effect of an 8-week exercise program among kindergarten children, with exercise resulting in increased P3 amplitudes and reduced P3 latencies [59]. However, this work has never been extended, to our knowledge, to preschool children, despite calls for its necessity [60].

While our study can provide insight into the influence of cardiorespiratory fitness on the cognitive abilities in preschool children, there are a few limitations to this study to consider. First, our assessment of cardiorespiratory fitness, the 6MWT, was based on a submaximal exercise test. Therefore, cardiorespiratory fitness was not directly measured. Furthermore, due to some participants choosing not to participate in some procedures, we had different sample sizes for each statistical test. In addition, this work represents a cross-sectional analysis precluding us from deriving any causal inferences between cardiorespiratory fitness and cognitive function among preschool-aged children. Therefore, it cannot be determined if cardiorespiratory fitness is driving better performance on cognitive tasks, or if children who perform better on cognitive tasks are more active and consequently have better cardiorespiratory fitness, or if there is a common underlying variable that was not captured in our measures. Additionally, we did not account for habitual physical activity patterns, attitudes and motivations towards physical activity, muscular strength or resistance training, as well as the time of day when testing occurred, each of which could potentially influence the findings. Data from the 6MWT and BMI also indicated a fairly fit and healthy sample, limited in racial and economic diversity. We anticipate cardiorespiratory fitness might explain a greater proportion of the variation in cognitive function among a more diverse group. However, we would expect that the positive relationship between 6MWT distance walked and cognitive function would remain, as studies in older children as well as those with greater BMI and different racial groups have also observed benefits of cardiorespiratory fitness for cognitive and brain health [61]. Nevertheless, the strengths of the present work included examining the influence of 6MWT distance walked after accounting for both adiposity and dietary factors, a common limitation of much of the current research in this area, as well as the introduction of a brain-based measure to extend neuro-imaging work into this population.

While several studies exist examining the relationship between cardiorespiratory fitness and neurocognitive abilities and academic achievement, much of this work has focused on school-aged children. The work presented here provides important evidence demonstrating that greater 6MWT distance is related to a wide range of cognitive function measures including academic achievement, intellectual abilities, cognitive flexibility, and attentional inhibition in children between 4–6 years. We also have provided some evidence that 6MWT distance has a tendency towards a relationship with neural signatures underlying selective attention, although more EEG work is needed to develop a more definitive understanding of this potential relationship. Given the prevalence of physical inactivity and poor fitness in youth across the globe, future experimental studies are needed to determine whether the benefits of physical activity interventions, evident in some studies in older children, are also evident in preschool-aged children.

## Figures and Tables

**Table 1:** Descriptive summary of main sample participant characteristics, diet, and task performance.

	Mean ± SD	Minimum	Maximum
Sex, Females/Males	25/34 (42% female)		
Age, years	5.0 ± 0.6	4.0	6.0
Race (*n* = 51)			
Asian, *n* (%)	6 (9.5)		
Black/African American, *n* (%)	3 (4.8)		
White or Caucasian, *n* (%)	34 (54)		
Mixed or Other, *n* (%)	8 (12.7)		
Household Income (*n* = 52)			
<10,000–40,000, *n* (%)	12 (19)		
41,000–80,000, *n* (%)	12 (19)		
≥81,000, *n*(%)	28 (44.4)		
BMI-for-age %ile	51.8 ± 33.7	0.1	99.9
Underweight, *n* (%)	4 (7)		
Normal or Healthy, *n* (%)	40 (70)		
Overweight, *n* (%)	4 (7)		
Obese, *n* (%)	9 (16)		
Visceral Adipose Tissue (*n* = 53)	113.6 ± 65.8	4.1	234.5
Total Healthy Eating Index-2015 (*n* = 55)	54.2 ± 14.2	24.3	92.0
Woodcock Johnson ECAD™ (*n* = 53)			
General Intellectual Ability	109.1 ± 14.2	76	141
Early Academic Skills	101.4 ± 14.3	72	138
Expressive Language	110.3 ± 16.0	76	143
Flanker Task (*n* = 48)			
Congruent Accuracy, %	65.4 ± 24.2	5	97.5
Congruent Reaction Time, ms	1246.8 ± 208.6	661.3	1629.5
Incongruent Accuracy, %	54.2 ± 24.2	7.5	100
Incongruent Reaction Time, ms	1293.8 ± 276.5	455.6	1781.8
Hearts and Flowers Task (*n* = 53)			
Homogeneous Accuracy, %	76.7 ± 17.3	20	100
Homogeneous Reaction Time, ms	945.0 ± 198.5	444.5	1318.5
Heterogeneous Accuracy, %	59.4 ± 21.6	10	95
Heterogeneous Reaction Time, ms	1137.5 ± 216.4	484.5	1537.4
6-Minute Walking Test (*n* = 59)			
Total Distance Walked, m	449.6 ± 82.0	240	600
Heart Rate (pre-test), bpm	97.3 ± 19.2	53	152
Heart Rate (post-test), bpm	132.3 ± 24.2	67	178
Oxygen Saturation (pre-test), %	97.6 ± 2.2	89	100
Oxygen Saturation (post-test), %	97.2 ± 2.0	92	100

SD, Standard Deviation; BMI, Body Mass Index; ECAD™, Early Cognitive and Academic Development Test.

**Table 2:** Regression analyses describing the association between six-minute walking test (6MWT) distance walked and academic skills and cognitive abilities among preschool-aged children.

		β	Coefficient *P*	*R* ^2^	Adjusted R^2^	Model *P*
Woodcock Johnson Early Cognitive and Academic Development Test (*n* = 53)	General Intellectual Ability			0.06	0.04	0.04
	6MWT Distance Walked	0.25 *	0.04			
	Early Academic Skills			0.13	0.09	0.05
	Visceral Adipose Tissue	−0.22	0.14			
	6MWT Distance Walked	0.25	0.09			
	Expressive Language			0.16	0.13	0.02
	Healthy Eating Index-2015	0.27 *	0.05			
	6MWT Distance Walked	0.30 *	0.04			
Flanker Task (*n* = 48)	Congruent Accuracy			0.24	0.18	<0.01
	Sex	0.29	0.02			
	General Intellectual Ability	0.25	0.04			
	6MWT Distance Walked	0.29 *	0.02			
	Congruent Reaction Time			0.14	0.08	0.04
	Age	−0.18	0.12			
	Sex	−0.26 *	0.04			
	6MWT Distance Walked	−0.16	0.14			
	Incongruent Reaction Time			0.07	0.05	0.04
	6MWT Distance Walked	−0.26 *	0.04			
Hearts and Flowers Task (*n* = 53)	Homogeneous Accuracy			0.24	0.21	<0.01
	Age	0.37 *	<0.01			
	6MWT Distance Walked	0.23 *	0.04			
	Homogeneous Reaction Time			0.17	0.14	0.01
	Age	−0.32 *	0.01			
	6MWT Distance Walked	−0.19	0.09			
	Heterogeneous Accuracy			0.43	0.40	<0.01
	Age	0.43 *	<0.01			
	General Intellectual Ability	0.29 *	0.01			
	6MWT Distance Walked	0.26 *	0.02			
	Heterogeneous Reaction Time			<0.01	<0.01	0.39
	6MWT Distance Walked	−0.04	0.39			

* *p* value < 0.05.

**Table 3:** Participant characteristics and performance indices for the subsample of children who completed the auditory oddball task.

	M	SD	Minimum	Maximum
Participant Characteristics (*n* = 26)				
Age, years	5.08	0.54	4.2	6.0
Sex, Females/Males	16, 10			
6-Minute Walking Test				
Total Distance Walked, m	465.50	68.39	289.7	580
Oddball Task Performance				
Accuracy for Targets (%)	75.26	18.23	27.5	97.5
Reaction Time to Targets (ms)	838.62	167.68	523.3	1115.3
Inverse Efficiency for Targets	12.20	5.12	6.1	28.0
Coefficient of Variation for Targets	0.38	0.11	0.21	0.64
ERP Indices				
Total Target Trials Used (of 40)	25.19	10.65	9.0	40.0
Artifact Free Trials	106.7	38.6	49.0	157.0
N2 Mean Amplitude Modulation	2.51	13.77	−25.0	34.4
N2 Peak Latency Modulation	−17.08	149.41	−312.0	266
P3 Mean Amplitude Modulation	4.17	14.80	−23.4	36.2
P3 Peak Latency Modulation	−38.08	142.73	−348.0	266.0

ERP, event-related potentials.

**Table 4:** Subsample partial correlation table examining auditory oddball, controlling for the variance of age and sex.

		Correlation with 6MWT Distance Walked	
Cognitive Task Performance		Pearson *r*	*p*-Value
	Accuracy for Targets (%)	0.075	0.754
	Reaction Time to Targets (ms)	0.388	0.061 †
	Target Inverse Efficiency	0.058	0.787
	Target Coefficient of Variation	−0.361	0.083 †
ERP Indices	N2 Mean Amplitude		
	Modulation	0.325	0.122
	Target	0.233	0.274
	Standard	−0.113	0.559
	N2 Peak Latency		
	Modulation	−0.231	0.277
	Target	−0.392	0.058 †
	Standard	−0.109	0.614
	P3 Mean Amplitude		
	Modulation	0.361	0.083 †
	Target	0.370	0.075 †
	Standard	−0.004	0.985
	P3 Peak Latency		
	Modulation	−0.093	0.665
	Target	0.212	0.320
	Standard	0.341	0.103

† *p* value < 0.1.

## Supplementary Materials

The following are available online at https://www.mdpi.com/2077-0383/10/4/584/s1, Figure S1: Bivariate correlations of significant outcomes from regression analysis, Table S1: Bivariate Correlations (single-tailed).
