# Supplementary material for: Six-Minute Walking Test Performance Relates to Neurocognitive Abilities in Preschoolers
Source: J Clin Med. 2021 Feb 4;10(4):584. doi: 10.3390/jcm10040584 (PMC7915798; doi:10.3390/jcm10040584)
Supplement: Supplementary file 1 [file jcm-10-00584-s001.pdf]

Table S1. Bivariate Correlations (single-tailed).

	1	2	3	4	5	6	7	8	9	10	11	12	13	14	15	16
1.6MWT Distance	-															
2. Age	0.292*	-														
3. Sex	-0.056	-0.067	-													
4.Household Income	0.048	0.140	-0.185	-												
5.Total HEI Score	0.140	-0.112	-0.321**	0.123	-											
6. VAT	-0.177	-0.245*	0.457**	-0.238	-0.365**	-										
7.General Intellectual Ability	0.250*	-0.040	-0.040	-0.016	0.222	-0.102	-									
8.Early Academic Skills	0.301*	0.016	-0.148	0.100	0.116	-0.257*	0.671**	-								
9.Expressive Language	0.247*	-0.033	0.013	0.287	0.291*	-0.239*	0.816**	0.591**	-							
10.Congruent Accuracy	0.236	0.190	0.306*	0.015	-0.086	0.177	0.285*	0.132	0.275*	-						
11.Congruent Reaction Time	-0.195	-0.241*	-0.247*	0.014	0.204	-0.103	0.058	0.100	0.038	-0.280*	-					
12.Incongruent Accuracy	0.113	0.258*	0.370**	0.145	-0.079	0.099	0.163	0.154	0.169	0.758**	-0.193	-				
13.Incongruent Reaction Time	-0.263*	-0.229	-0.053	0.087	0.177	-0.025	0.060	0.162	0.081	-0.027	0.710**	0.044	-			
14.Homogenous Accuracy	0.337**	0.446**	0.074	0.025	0.046	-0.066	0.204	0.191	0.216	0.436**	0.066	0.462**	0.238	-		
15.Homogenous Reaction Time	-0.283*	-0.394**	-0.057	0.024	-0.077	0.082	-0.001	0.043	0.009	-0.143	0.533**	-0.186	0.497**	-0.231*	-	
16.Heterogenous Accuracy	0.397**	.500**	0.043	-0.043	0.078	-0.211	0.323*	0.244*	0.308*	0.544**	-0.017	0.474**	0.083	0.524**	-0.214	-
17.Heterogenous Reaction Time	-0.040	0.014	0.125	-0.182	0.000	-0.012	0.228	0.173	0.165	0.135	0.336*	0.078	0.258*	0.203	0.506**	0.317**

$p < 0.05$; ** $p < 0.01$; 6MWT- six minute walking; HEI-healthy eating index; VAT- visceral adipose tissue.

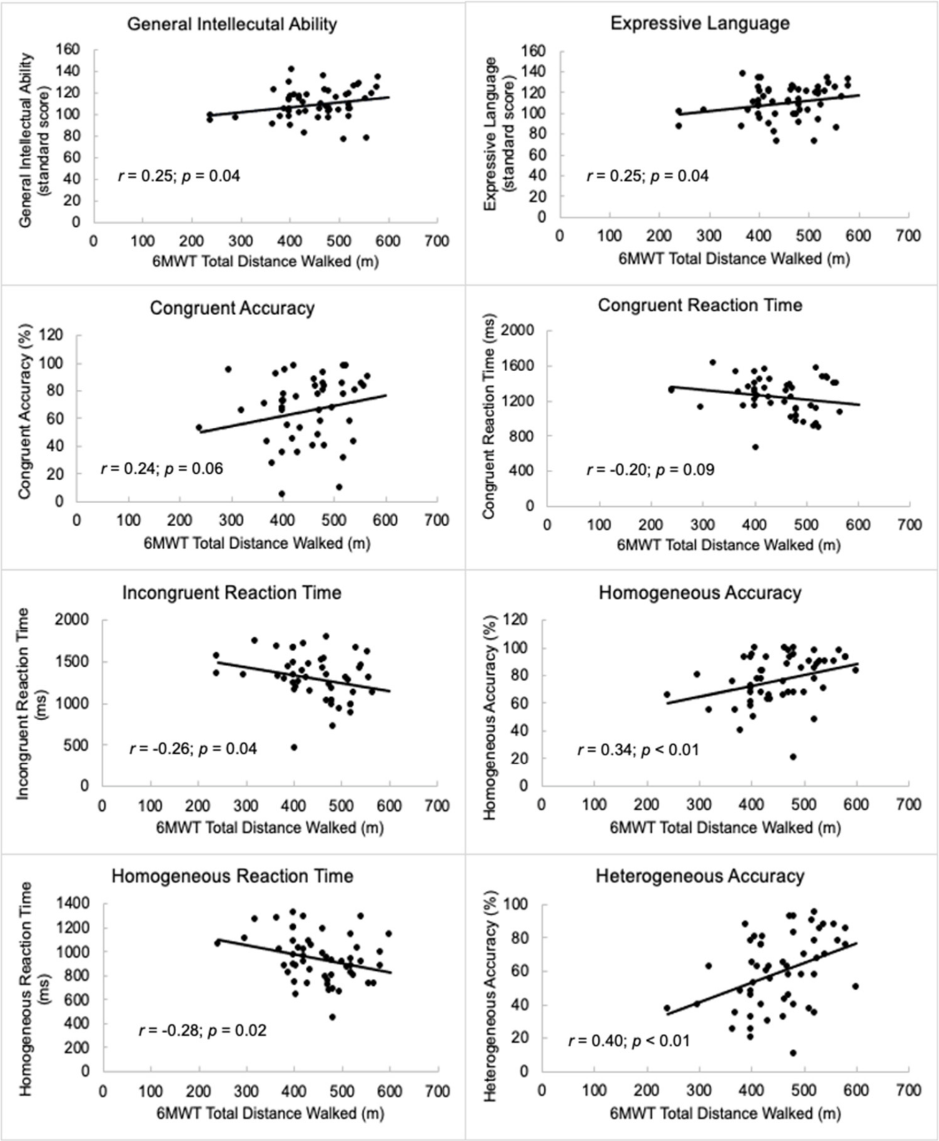

Figure S1. Bivariate correlations of significant outcomes from regression analysis.
